# Malaria-Related Psychosocial Factors, Past Antenatal Care–Seeking Behaviors, and Future Antenatal Care–Seeking Intentions by Maternal Age in Malawi and Democratic Republic of the Congo

**DOI:** 10.4269/ajtmh.23-0069

**Published:** 2023-06-26

**Authors:** Bolanle Olapeju, Michael Bride, Julie R. Gutman, Jessica K. Butts, Ashley Malpass, Anna McCartney-Melstad, Lynn M. Van Lith, Katie Rodriguez, Susan Youll, Nyanyiwe Mbeye, Ferdinand Ntoya, Sosten Lankhulani, Florence Mpata, Stella Babalola

**Affiliations:** ^1^ Uniformed Services University of the Health Sciences, Bethesda, Maryland;; ^2^ Johns Hopkins Center for Communication Programs, Baltimore, Maryland;; ^3^ Malaria Branch, Center for Global Health, Centers for Disease Control and Prevention, Atlanta, Georgia;; ^4^ U.S. President’s Malaria Initiative, Malaria Branch, U.S. Centers for Disease Control and Prevention, Atlanta, Georgia;; ^5^ U.S. President’s Malaria Initiative, United States Agency for International Development, Washington, District of Columbia;; ^6^ Department of Epidemiology and Biostatistics, Kamuzu University of Health Sciences, Lilongwe, Malawi;; ^7^ U.S. President’s Malaria Initiative, United States Agency for International Development, Kinshasa, Democratic Republic of the Congo;; ^8^ National Malaria Control Program, Ministry of Health, Lilongwe, Malawi

## Abstract

Young women in sub-Saharan Africa are a group at increased risk for malaria in pregnancy. Early antenatal care (ANC) seeking makes it more likely that women will receive the recommended doses of intermittent preventive treatment of malaria in pregnancy. This study used data from national Malaria Behavior Surveys conducted in Malawi and the Democratic Republic of the Congo (DRC) in 2021 to explore the association between intention to attend ANC in the first trimester for a future pregnancy (early ANC intention) and psychosocial factors among women aged 15–49 years. Eight psychosocial factors related to ANC and based on the ideation model were included, including knowledge, attitudes, and self-efficacy. The study used multivariable logistic regression models controlling for demographic characteristics to evaluate associations between early ANC intention and the individual ideational factors and the composite measure. Analysis included 2,148 women aged 15–49 years (Malawi: 827, DRC: 1,321). Antenatal care ideation was lower among young (aged 15–20 years) than among older (aged 21–49 years) women in Malawi. Young mothers with higher ANC ideation were more likely to intend to attend ANC early in their next pregnancy in both countries. Specific ideational factors associated with intention to attend ANC early varied by country and included positive attitudes, knowledge of ANC, and positive self-efficacy. In Malawi and the DRC, youth-friendly social and behavior change interventions to increase ANC-related ideation could increase future early ANC attendance among young women to improve malaria and birth outcomes.

## INTRODUCTION

The WHO’s antenatal care (ANC) guidelines recommend a minimum of eight contacts with a health provider, with first contact before 12 weeks’ gestation. Each contact is an opportunity for comprehensive care, including screening for and treatment of complications and informational sessions on a healthy pregnancy.^1^ In malaria-endemic areas, ANC visits often include an additional focus on this illness because pregnant women have a higher risk of infection than nonpregnant women.^2^ Malaria in pregnancy (MIP) contributes to preterm birth and low birth weight, which increases the risk of morbidity, including cognitive and social developmental delays and mortality.^3^ Antenatal care attendance in the first trimester of pregnancy (early ANC) is associated with a greater likelihood of reducing the risk of MIP as well as associated adverse outcomes through the provision of insecticide-treated nets (ITNs), intermittent preventive treatment during pregnancy (IPTp) for malaria with sulfadoxine-pyrimethamine starting in the second trimester, and other essential screening and treatments.^4^

Malaria is endemic in both Malawi and the Democratic Republic of the Congo (DRC), with annual incidence rates in 2020 of 229 and 324 per 1,000 population, respectively.^5^ The maternal mortality ratio is estimated to be 439 per 100,000 live births in Malawi and 473 per 100,000 live births in the DRC.^6^ National policy for ANC and IPTp is similar in both countries with some nuanced differences; both countries recommend at least four ANC visits with provision of a free ITN at first ANC and initiation of IPTp in the 14th week. In Malawi, the national policy goes further to encourage pregnant women to attend a minimum of eight ANC visits, starting as soon as they know they are pregnant.^7^ In both countries, access to health services represents a barrier to ANC attendance, and the proportion of the total population living in rural areas is 83% in Malawi and 54% in the DRC. Neither country currently has an operational policy of community-based delivery of IPTp, though the DRC will likely officially adopt this policy in the near future. Both countries have focal initiatives of community outreach to encourage women to attend ANC, including follow-up visits for existing ANC clients. In Malawi, all ANC appointments and medications are free at public facilities and clinics, and many private facilities also offer free services through a service agreement with the government. In the DRC medications are free, but ANC clients must cover the cost of associated laboratory fees and the ANC booklet where services are recorded. Therefore, the more ANC visits a woman makes during her pregnancy, the more she must pay out of pocket for her care, which represents another potential barrier to ANC attendance.

Despite recommendations for four (DRC) or eight (Malawi) visits, the proportion of pregnant women attending at least four ANC visits is relatively low, at 51% in Malawi and 43% in the DRC.^8^^,^^9^ Intermittent preventive treatment during pregnancy uptake differs significantly between Malawi and the DRC, with 41% of women in Malawi taking at least three doses of IPTp in their past pregnancy compared with only 13% in the DRC.^8^^,^^9^ Early ANC attendance has typically been low in both Malawi and the DRC, with only 24% and 17% of pregnant women attending ANC in their first trimester in Malawi and the DRC, respectively.^8^^,^^9^

Adolescent pregnancies account for almost 20% of pregnancies in sub-Saharan Africa, yet this group receives little attention in the relevant literature.^10^^,^^11^ In Malawi and the DRC, more than 60% and 50% of women, respectively, have had a child by age 20 years.^8^^,^^9^ Compared with older women, adolescents have a higher risk of MIP. Although this is partly due to primigravity,^12^ research shows that pregnant adolescents attend fewer ANC visits and report lower quality of ANC services in addition to social stigma.^13^^–^^15^ The extent to which age influences ANC-seeking intentions is unclear, presenting unique opportunities to explore how models of communication and behavior change can be applied to this particularly vulnerable group to optimize their pregnancy outcomes.

The ideation model of strategic communication and behavior change refers to how views and ideas (ways of thinking) are developed and disseminated within communities through communication and social interaction.^16^^,^^17^ The model incorporates intermediate cognitive, emotional, and social factors from various behavioral theories and includes ideational factors that act synergistically to influence behavior.^18^ Ideational factors demonstrated as correlates of malaria-related and/or ANC behavior include attitudes toward ANC, role in decision-making, interpersonal communication, knowledge of malaria and its risks, perceived threat of malaria, and self-efficacy to prevent malaria.^18^^–^^23^ Ideational factors also influence intention, an important and proximal determinant of behavior.^24^ Although studies have shown the link between ideational factors and intention to use ITNs^19^ or receive IPTp,^25^ the relationship between ideation and the intention to seek ANC early, particularly among young mothers, remains unclear.

Exploring young mothers’ ANC-related ideation and past behaviors could inform behavior change approaches and improve early ANC attendance among youth as a vulnerable group. This study explores ideational factors and past behaviors related to women’s intention to attend early ANC among young mothers compared with older mothers in Malawi and the DRC.

## MATERIALS AND METHODS

Data derived from the Malaria Behavior Survey (MBS), a multicounty community-level survey informed by the ideation model designed to assess ideational and other factors associated with malaria-related behaviors using a standardized methodology, were used in this analysis.^22^ Malawi and the DRC were selected for analysis given the similar timeline of study implementation (DRC: March–April 2021; Malawi: May–June 2021).

### Data collection.

Cross-sectional surveys, representative of Malawi regions and DRC provinces, were conducted in both countries using multistage cluster random sampling and probability proportional to size approaches. After cluster selection, all households within the cluster were listed, and eligible households were randomly selected. Households with at least one woman aged 15–49 years were eligible to participate. In each selected household, a household representative, as well as all women between ages 15 and 49 years who agreed to participate, were surveyed. This analysis focused on women who reported a live birth in the 2 years prior to the survey and intended to have another pregnancy in the future. Data collection took place in the months at the end of or after the rainy seasons, specifically in the DRC from March–April 2021 and in Malawi from May–June 2021.

### Variables.

The outcome variable for this analysis was the intention to attend ANC early in a future pregnancy. The outcome variable was based on the following survey question: “At what month in your pregnancy would you intend to go for your first antenatal visit?”

Independent variables included past early ANC behavior, sociodemographic characteristics, and ideational factors. Ideational factors explored in the surveys assessed respondents’ ideation toward malaria in general, as well as their ideation specific to MIP. These variables included perceived severity, perceived susceptibility, knowledge about malaria and prevention of MIP, perceived self-efficacy to go to ANC early, perceived response efficacy of IPTp, favorable perceptions toward health facilities and providers, perceived supportive community norms (if ANC attendance or IPTp uptake is considered common in their community), and involvement in decision-making related to ANC. Aside from knowledge, ideational factors were assessed using a series of Likert scale questions informed by the 2017 WHO Roll Back Malaria, Malaria Behavior Change Communication Indicator Reference Guide.^26^ Knowledge variable calculations were based on correct/incorrect responses to objective questions about signs, cause, and prevention of malaria, as well as timing and frequency of ANC visits.

Sociodemographic characteristics used in the analysis included age (< 20 years versus ≥ 20 years), region (north, central, and southern for Malawi; north, west, Kasai, and Great East for the DRC), residency (rural or urban), level of education completed (primary or not), marital status (married or not), wealth quintile (lower three quintiles or upper two, based on a principle component analysis of physical characteristics, such as the type of roof, floor, water infrastructure used, and assets owned by a household), parity (one versus more), exposure to malaria prevention or treatment messages (in the past 6 months in Malawi and 1 year in the DRC), weekly radio listenership (yes or no), weekly television viewership (yes or no), and mobile phone ownership (yes or no). Descriptions of all items used to measure ideation are available in Supplemental Table 1.

### Data analysis.

Data management and analysis were done using Stata version 17 (Stata Corporation, College Station, TX). The data were weighted using the svyset command in STATA to make the data representative of the national study population and to account for the multistage study design that relied on probability proportional to size. Each country was analyzed and weighted separately from each other. Only women who had a previous pregnancy in the past 2 years and intended to have another pregnancy were included in the analysis. Each question that assessed an ideational factor was re-coded based on whether the response favored the factor or not. For example, women who agreed that “every case of malaria could potentially lead to death” received a score of 1, whereas those who disagreed were scored −1 and those who were unsure were ascribed a score of 0. Scores from all questions related to a given ideational factor were summed to produce a net score, which was then dichotomized based on the median. Finally, an overall ideation score was created by summing all the ideational factors, thus ranging from 0 to 8 points. Analytical methods included χ^2^ tests of association, violin plots, and unadjusted and multivariable logistic regressions. The χ^2^ tests explored the association between the various outcomes and ideational factors with maternal age. Violin plots assessed the distribution of the overall ideation scores by maternal age. Unadjusted and multivariable logistic regression models were created for each individual country and explored factors associated with the intention to attend ANC early in a future pregnancy, stratified by age. Covariates included region, residence, education, wealth, marital status, parity and exposure to malaria messages, overall ideation score or specific ideational factors, and early attendance at ANC in past pregnancy. A priori consideration of the ideational model factors guided the exploration of all ideational factor variables in the multivariable regression.

## RESULTS

Of the total samples of 4,181 women in Malawi MBS and 6,034 women in the DRC MBS, 827 and 1,321 women, respectively, were included based on previous pregnancy in the past 2 years and intention to have another child. Roughly one-third (28.2%) and one-fifth (17.6%) were 20 years old or younger in Malawi and the DRC, respectively (Table 1). Overall, rural residence, parity, and radio listenership were higher in Malawi, whereas the DRC had higher levels of education, wealth, exposure to messages, and TV viewership. In both countries, young women were more likely to be primiparous but less likely to be married, exposed to malaria messages, watch TV, or own a mobile phone.

**Table 1:** Description of study population by maternal age

Characteristics (%)	Malawi				DRC			
	≤ 20 years (*N* = 234)	> 20 years (*N* = 593)	Total (*N* = 827)	*P* value	≤ 20 years (*N* = 233)	> 20 years (*N* = 1,088)	Total (*N* = 1,321)	*P* value
Region				0.106				0.001
Northern	38	32	34	–	–	–	–	–
Central	24	33	30	–	–	–	–	–
Southern	39	36	37	–	–	–	–	–
North	–	–	–	–	30	15	18	–
West	–	–	–	–	31	46	44	–
Kasai	–	–	–	–	14	16	16	–
Great East	–	–	–	–	25	23	23	–
Rural residence	94	90	91	0.278	58	44	47	0.248
Education > primary	50	47	48	0.766	73	81	80	0.691
Marital status married or cohabitating	88	93	91	0.005	77	87	86	< 0.001
Wealth quintile upper two	28	43	38	0.184	57	43	54	< 0.001
Parity, one	89	33	51	< 0.001	58	21	27	< 0.001
Exposure to malaria message	11	31	24	0.005	29	34	33	0.07
Listens to radio weekly	56	43	48	0.001	35	38	37	0.035
Watches TV weekly	3	9	7	0.015	25	39	37	0.001
Household has mobile phone	20	36	31	< 0.001	15	34	31	< 0.001

DRC = Democratic Republic of the Congo. *P* values denote differences between age groups.

### ANC outcomes and future intentions by maternal age.

Table 2 describes previous ANC outcomes and future intentions by age group. Slightly more than one-third of women in both countries attended ANC during the first trimester of their most recent past pregnancy, whereas one-half or more attended it during their second trimester. Young women in the DRC (33%) were slightly less likely to have attended ANC early than those older than 20 years (39%). The majority of women in Malawi (94%) had received at least one dose of IPTp in their past pregnancy compared with three-quarters of women in the DRC. Just under one-half of young women in Malawi (48%) had received three or more doses of IPTp in their past pregnancy compared with 60% of the older women; in the DRC, 39% of younger women and 44% of older women received three doses. Three-quarters of women in Malawi overall intended to attend ANC in the first trimester of their next pregnancy compared with just over one-half of women in the DRC, whereas the majority of women (MWI: 98%, DRC: 95%) in both countries intended to get IPTp in their next pregnancy.

**Table 2:** ANC outcomes and future intentions by maternal age

ANC outcomes and future intentions	Malawi				DRC			
	≤ 20 years (*N* = 234)	> 20 years (*N* = 593)	Total (*N* = 827)	*P* value	≤ 20 years (*N* = 233)	> 20 years (*N* = 1,088)	Total (*N* = 1,321)	*P* value
Past pregnancy outcomes								
Month of first ANC visit attended								
Early (0–3)*	31	37	35	0.928	33	39	38	0.017
4–6	64	52	56		52	50	50	
7–9	3	7	6		7	7	7	
Number of IPTp doses obtained								
At least one	95	94	94	0.930	79	79	79	0.788
At least two	86	84	84	0.630	67	65	65	0.533
Three or more	48	60	56	0.818	39	44	43	0.305
Future pregnancy intentions								
Early* first ANC visit	77	71	76	0.321	59	59	58	0.481
Any IPTp uptake	97	98	98	0.745	95	97	95	0.481

ANC = antenatal care; DRC = Democratic Republic of the Congo; IPTp = intermittent preventive treatment during pregnancy. *P* value denotes differences between age groups.

* Early ANC is defined as within 0–3 months of pregnancy.

### Ideation by maternal age.

Table 3 describes the prevalence of each of the ideational factors by country and age group. Prevalent (> 60%) ideational factors in both countries included perceived severity of malaria, positive perceptions of health care workers, perceived self-efficacy to go to ANC early, and positive community norms around ANC. Perceived susceptibility to malaria was less prevalent (< 40%) across both countries.

**Table 3:** Malaria ideation by maternal age

Ideational factors (%)	Malawi				DRC			
	≤ 20 years (*N* = 234)	> 20 years (*N* = 593)	Total (*N* = 827)	*P* value	≤ 20 years (*N* = 233)	> 20 years (*N* = 1,088)	Total (*N* = 1,321)	*P* value
Knowledge of malaria	48	59	55	0.249	79	81	81	0.118
Knowledge of ANC	59	51	53	0.952	40	36	36	0.139
Positive attitudes toward ANC	72	65	67	0.614	33	35	35	0.296
Perceived susceptibility to malaria	26	32	30	0.688	34	32	33	0.248
Perceived severity of malaria	87	90	89	0.045	59	61	60	0.755
Self-efficacy related to ANC	97	96	96	0.662	75	80	79	0.323
Positive perceptions of health care workers regarding MIP	63	78	73	0.972	92	90	90	0.799
Supportive community norms related to ANC	79	80	80	0.619	74	74	74	0.497

ANC = antenatal care; DRC = Democratic Republic of the Congo; MIP = malaria in pregnancy. *P* value denotes differences between age groups.

### Violin plots of ANC ideation by maternal age.

**Figure 1.**
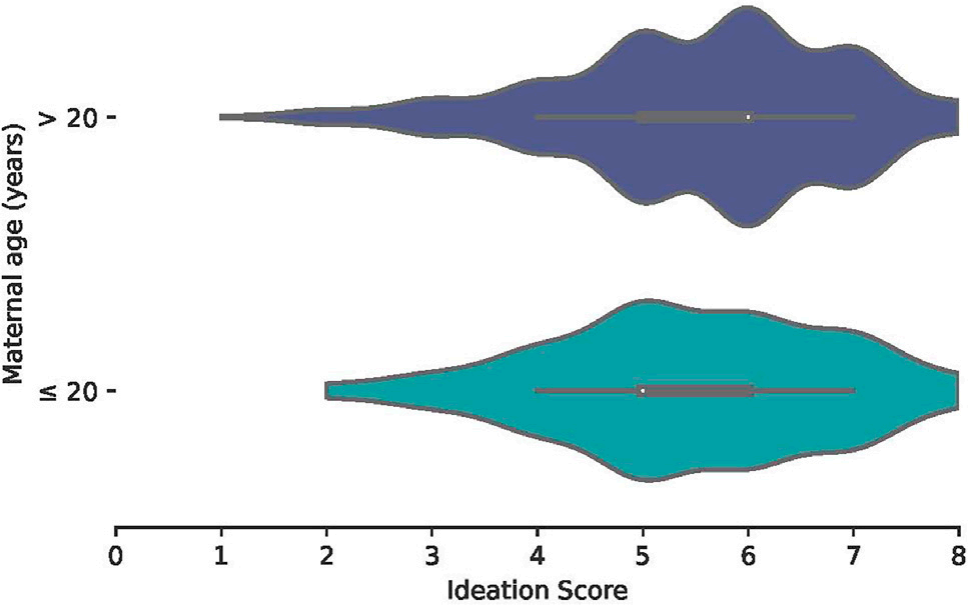
Violin plot of ANC ideation by maternal age, Malawi. Antenatal care ideation was scored on a scale of 0–8 for each ideational factor. The white dot represents the median score while the box represents the interquartile range and the whiskers represent the minimum and maximum values. ANC = antenatal care.

**Figure 2.**
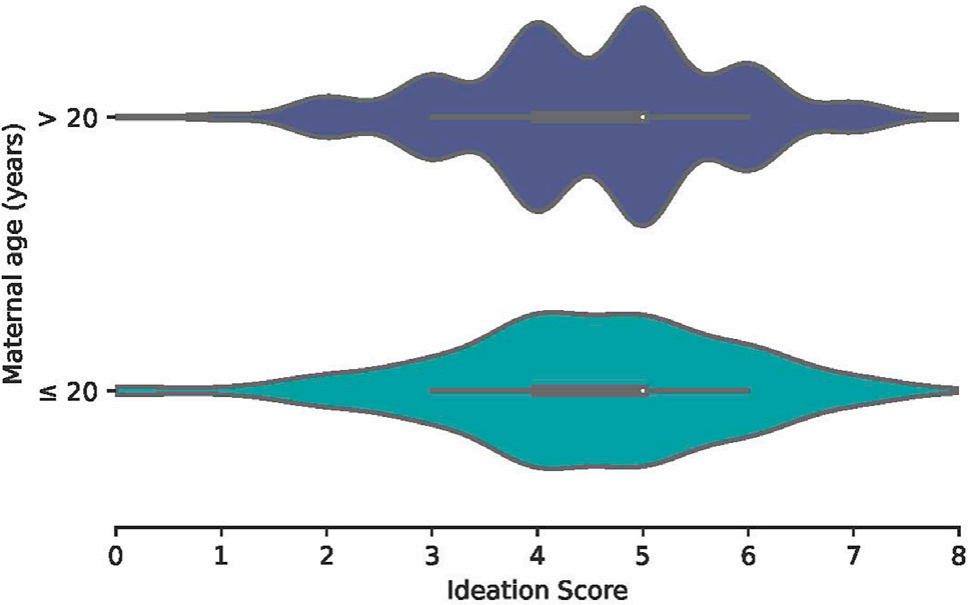
Violin plot of ANC ideation by maternal age, the DRC. Antenatal care ideation was scored on a scale of 0–8 for each ideational factor. The white dot represents the median score while the box represents the interquartile range and the whiskers represent the minimum and maximum values. ANC = antenatal care; DRC = Democratic Republic of the Congo.

Figures 1 and 2 show violin plots of ANC ideation score (range: 0–8) by maternal age across countries. Although mean scores were similar between age groups in the DRC (≤ 20 years mean: 4.53, SD: 4.40–4.67; > 20 years mean: 4.54, SD: 4.40–4.67), mean scores were lower among young mothers (mean: 5.29, SD: 5.12–5.47) than among older mothers (mean: 5:50, SD: 5.35–5.61) in Malawi. Malawi also had higher ideation scores overall than the DRC.

### Ideational factors associated with intention to attend ANC in the first trimester of a future pregnancy by maternal age.

Among young women with a prior pregnancy, factors associated with intention to attend ANC in the first trimester in a future pregnancy in either Malawi or the DRC included knowledge of ANC, positive attitudes toward ANC, and perceived self-efficacy toward ANC (Table 4).

**Table 4:** Intention to attend ANC within the first trimester in future pregnancy

Specific ideational factors	Malawi		DRC	
	≤ 20 years (*N* = 234)	> 20 years (*N* = 593)	≤ 20 years (*N* = 233)	> 20 years (*N* = 1,088)
Knowledge of malaria	1.65 (0.86–3.16)	1.27 (0.83–1.93)	1.25 (0.58–2.71)	1.65† (1.13–2.39)
Knowledge of ANC	3.39‡ (1.69–6.81)	2.56‡ (1.63–4.02)	1.63 (0.81–3.32)	1.80‡ (1.32–2.46)
Attitudes toward ANC	2.28* (1.18–4.41)	1.24 (0.81–1.91)	1.79 (0.83–3.89)	1.69† (1.23–2.33)
Perceived susceptibility to malaria	1.10 (0.55–2.18)	1.36 (0.87–2.12)	1.01 (0.50–2.02)	0.86 (0.63–1.18)
Perceived severity of malaria	2.60 (0.97–6.95)	0.72 (0.32–1.62)	0.72 (0.35–1.46)	1.13 (0.84–1.52)
Self-efficacy related to ANC	1.04 (0.29–3.67)	2.54* (1.13–5.75)	5.18† (1.92–14.00)	2.41‡ (1.59–3.66)
Perceptions of health care workers in regard to MIP	1.92 (0.87–4.26)	1.39 (0.83–2.34)	2.64 (0.80–8.70)	1.27 (0.78–2.07)
Supportive community norms related to ANC	0.93 (0.45–1.92)	1.15 (0.72–1.84)	1.00 (0.48–2.10)	0.60† (0.43–0.83)

ANC = antenatal care; MIP = malaria in pregnancy. Data are adjusted odds ratio (95% CI); reference group is the null (no category).

* *P* < 0.05.

† *P* < 0.01.

‡ *P* < 0.001.

Among older women, knowledge of both malaria and ANC, as well as positive attitudes and self-efficacy toward ANC, were all positively associated with intention to attend ANC in the first trimester in either the DRC or Malawi, whereas positive community ANC norms were negatively associated in the DRC.

In multivariable logistic regression models including the composite ideation score (instead of individual ideational factors), overall ANC ideation was positively associated with the intention to attend ANC within the first trimester in both age groups and both countries, as well as with past behaviors (attending ANC in the first trimester of their last pregnancy), which was significant in Malawi for older women > 20 years old and both DRC age groups (Supplemental Table 2). There were also statistically significant regional differences in the DRC.

## DISCUSSION

Using data from the MBS, this study explored ideational factors related to ANC attendance during the first trimester of pregnancy, with a focus on women aged 15–20 years, an understudied population at higher risk of MIP. This analysis suggests that ideational factors that could influence intentions to seek ANC care early may vary depending on the age group of the mother and, therefore, could benefit from more nuanced and targeted behavior change approaches to improving MIP outcomes. Not only were the significant ideational factors different between younger and older participants, but they were also different among these groups in different countries, confirming the importance of country context as well as age.

Despite the fact that adolescents make up a substantial proportion of childbearing women, research has not paid much attention to the specific needs of adolescent mothers and how they may differ from those of older women. This is particularly crucial, given that both young age and first pregnancy are independent risk factors for malaria infection in pregnancy and that these data show that young mothers are less likely to present for ANC early in pregnancy and are less likely to receive the recommended doses of IPTp.^2^

The need to improve malaria-related ideation among young and older mothers is a key takeaway from this analysis, as ideation is a key predictor of intention and thus behavior. Malaria-related ideation can be improved through a variety of youth-targeted social and behavior change (SBC) interventions. Pregnant youth and those who influence them are a special audience with unique needs. Even within the youth population studied here, the concerns of a 15-year-old vary greatly from those of a 20-year-old, highlighting the diversity within this group and requiring tailored SBC approaches to any given country context. A great deal of research highlights the impact of positive youth development approaches in general; these may be applied to encouraging future ANC attendance and improving malaria prevention.^27^^,^^28^ Compared with their older counterparts, more young pregnant women in this analysis were unmarried in both countries, were in lower wealth quintiles in the DRC, and had less exposure to malaria-related messages that support early ANC in Malawi. National malaria control programs and SBC practitioners are well placed to address the specific needs of younger pregnant women to avoid missed opportunities for client interaction and IPTp administration while ensuring equitable reach to those often overlooked in programming.

There is an additional need to explore channels and platforms that are appropriate for engaging youth. Previous interventions focused on education, and although several countries are making strides in improving access to education for pregnant youth, many may still be unable to continue attending school once they are noticeably pregnant, limiting the effectiveness of school-based interventions.^29^ Further formative research on optimal ways to reach pregnant youth should be done prior to deciding the channels for SBC activities. These activities should include targeted messaging centered around building the factors that comprise malaria ideation. A process for co-developing approaches that meet the complex needs of youth should, as a principle, include young people. Between Malawi and the DRC, there are marked differences in media access, previous malaria message exposure, and resources, which are important considerations for implementation. Malawi and the DRC also had different significant determinants for intention, such as attitudes for Malawi youth, which highlights the importance of focusing on the specific factors that most impact malaria ideation and utilization of preventive behaviors prior to designing an SBC intervention for this special audience.

An important point of interest is the negative association of community ANC norms and intention to attend ANC in the first trimester among older women in the DRC alone. This negative association was not initially expected given the theoretical framework but has been noted anecdotally, not only in regard to ANC but in care seeking for fever as well; it is presumed to be due to several factors including the unique health care context of the DRC, in which additional barriers such as high perceived costs might outweigh community norms, as well as perhaps a lower importance on community norms in regard to important behaviors.

Although the use of data from the MBS, a unique nationally representative survey that allows for robust and in-depth investigations of demographic and psychosocial correlates of malaria-related behaviors across different countries, can provide a useful initial assessment of how ideational factors differ between younger and older women in Malawi and the DRC, it contains several limitations. The overall proportion of women in the “young” group was relatively small, just under 30% in Malawi and less than 20% in the DRC. This limited the statistical power for more in-depth analysis, particularly for the DRC. Social desirability bias may have caused women to respond affirmatively to questions, even if this did not reflect their true feelings. In addition, translation of the questions from English to French or local languages may have altered nuances of the questions, which might have influenced the response. Because of the survey design, only women with prior pregnancies and intentions to have additional pregnancies were included; however, when considering how to best influence young women to attend ANC, considering the ideations and behaviors of women who have not been pregnant previously and whether they require different messaging or channels to optimally target them for early and frequent ANC, may be most critical.

Given the substantial differences between Malawi and the DRC, these findings cannot be generalized as seen to other countries; rather, these observations should prompt assessment of youth-related ideational and behavioral issues in additional contexts, as they may vary between and within countries. Armed with information about the most influential factors shaping young pregnant women’s care-seeking behavior, ministries of health and SBC practitioners across the globe may more effectively address the unique needs of this segment of the population and capitalize on an important opportunity to increase early ANC, prevent MIP, and improve pregnancy outcomes more generally. Although generating demand for services among this group is essential, ensuring that their experience of care is youth friendly and nonstigmatizing once they arrive is equally important.

## Supplemental Material


Supplemental materials

## References

[1] World Health Organization , 2014. *WHO Policy Brief for the Implementation of Intermittent Preventive Treatment of Malaria in Pregnancy Using Sulfadoxine-Pyrimethamine (IPTp-SP)*. Available at: file:///C:/Users/judym/Downloads/WHO-HTM-GMP-2014.4-eng.pdf. Accessed July 6, 2022.

[2] Rogerson SJ Desai M Mayor A Sicuri E Taylor SM van Eijk AM, 2018. Burden, pathology, and costs of malaria in pregnancy: new developments for an old problem. Lancet Infect Dis 18: e107–e118. 29396010 10.1016/S1473-3099(18)30066-5

[3] Chua CLL Hasang W Rogerson SJ Teo A, 2021. Poor birth outcomes in malaria in pregnancy: recent insights into mechanisms and prevention approaches. Front Immunol 12: 621382. 33790894 10.3389/fimmu.2021.621382 PMC8005559

[4] Apanga PA Kumbeni MT Chanase M-AW, 2022. The association between early antenatal care and intermittent preventive treatment of malaria in pregnancy in sub-Saharan Africa: effect modification by planned pregnancy status. Ann Glob Health 88: 4. 35087704 10.5334/aogh.3550 PMC8757383

[5] World Health Organization, Global Health Observatory Data Repository/World Health Statistics *Incidence of Malaria*. Available at: https://data.who.int/countries/454. Accessed July 6, 2022.

[6] WHO , 2019. Trends in Maternal Mortality 2000 to 2017: Estimates by WHO, UNICEF, UNFPA, World Bank Group and the United Nations Population Division. Geneva, Switzerland: World Health Organization.

[7] National Malaria Control Programme. Revised Malaria Strategic Plan, 2020.

[8] Enquête par grappes à indicateurs multiples , 2017–2018. rapport de résultats de l’enquête. L’Institut National de la Statistique Kinshasa, République Démocratique du Congo. Available at: https://mics-surveys-prod.s3.amazonaws.com/MICS6/West%20and%20Central%20Africa/Congo%2C%20Democratic%20Republic%20of%20the/2017-2018/Survey%20findings/Congo%2C%20Democratic%20Republic%20of%20the%2C%202017-18%20MICS%20SFR_French.pdf. Accessed July 6, 2022.

[9] National Statistical Office/Malawi and ICF , 2017. Malawi Demographic and Health Survey 2015–16. Zomba, Malawi: National Statistical Office and ICF.

[10] Kassa GM Arowojolu AO Odukogbe AA Yalew AW, 2018. Prevalence and determinants of adolescent pregnancy in Africa: a systematic review and meta-analysis. Reprod Health 15: 195. 30497509 10.1186/s12978-018-0640-2 PMC6267053

[11] Pell C Straus L Andrew EVW Meñaca A Pool R, 2011. Social and cultural factors affecting uptake of interventions for malaria in pregnancy in Africa: a systematic review of the qualitative research. PLoS One 6: e22452. 21799859 10.1371/journal.pone.0022452 PMC3140529

[12] Brabin BJ Warsame M Uddenfeldt-Wort U Dellicour S Hill J Gies S, 2008. Monitoring and evaluation of malaria in pregnancy – developing a rational basis for control. Malar J 7: S6. 19091040 10.1186/1475-2875-7-S1-S6 PMC2604870

[13] Owolabi OO Wong KLM Dennis ML Radovich E Cavallaro FL Lynch CA Fatusi A Sombie I Benova L, 2017. Comparing the use and content of antenatal care in adolescent and older first-time mothers in 13 countries of west Africa: a cross-sectional analysis of demographic and health surveys. Lancet Child Adolesc Health 1: 203–212. 30169169 10.1016/S2352-4642(17)30025-1

[14] Hackett K Lenters L Vandermorris A LaFleur C Newton S Ndeki S Zlotkin S, 2019. How can engagement of adolescents in antenatal care be enhanced? Learning from the perspectives of young mothers in Ghana and Tanzania. BMC Pregnancy Childbirth 19: 184. 31122199 10.1186/s12884-019-2326-3 PMC6533671

[15] Pell C , 2013. Factors affecting antenatal care attendance: results from qualitative studies in Ghana, Kenya and Malawi. PLoS One 8: e53747. 23335973 10.1371/journal.pone.0053747 PMC3546008

[16] Kincaid DL, 2000. Mass media, ideation, and behavior. Communic Res 27: 723–763.

[17] Kincaid DL, 2000. Social networks, ideation, and contraceptive behavior in Bangladesh: a longitudinal analysis. Soc Sci Med 50: 215–231. 10619691 10.1016/s0277-9536(99)00276-2

[18] Monroe A Olapeju B Moore S Hunter G Merritt AP Okumu F Babalola S, 2021. Improving malaria control by understanding human behaviour. Bull World Health Organ 99: 837–839. 34737477 10.2471/BLT.20.285369 PMC8542269

[19] Asingizwe D Poortvliet PM Koenraadt CJM van Vliet AJH Ingabire CM Mutesa L Leeuwis C, 2019. Role of individual perceptions in the consistent use of malaria preventive measures: mixed methods evidence from rural Rwanda. Malar J 18: 270. 31395048 10.1186/s12936-019-2904-x PMC6686450

[20] Do M Babalola S Awantang G Toso M Lewicky N Tompsett A, 2018. Associations between malaria-related ideational factors and care-seeking behavior for fever among children under five in Mali, Nigeria, and Madagascar. PLoS One 13: e0191079. 29370227 10.1371/journal.pone.0191079 PMC5784922

[21] Ricotta EE Boulay M Ainslie R Babalola S Fotheringham M Koenker H Lynch M, 2015. The use of mediation analysis to assess the effects of a behaviour change communication strategy on bed net ideation and household universal coverage in Tanzania. Malar J 14: 15. 25603882 10.1186/s12936-014-0531-0 PMC4308934

[22] Kumoji EK Awantang GN Toso M Kamara D Bleu T Lahai W Sillah-Kanu M Dosso A Achu D Babalola S, 2022. Ideational factors associated with net care behaviour: a multi-country analysis. Malar J 21: 53. 35177086 10.1186/s12936-022-04053-5 PMC8851768

[23] Okedo-Alex IN Chizoba-Akamike I Ezeanosike OB Uneke CJ, 2019. Determinants of antenatal care utilisation in sub-Saharan Africa: a systematic review. BMJ Open 9: e031890. 10.1136/bmjopen-2019-031890 PMC6797296 31594900

[24] Ajzen I, 1991. The theory of planned behavior. Organ Behav Hum Decis Process 50: 179–211.

[25] Nsutier OK Bongo GN Tshiama RC Kibongo CK Kanika JM Mukuandu L Babintu B, 2018. Determinants of pregnant women intention to respect the optimal timeframe for intermittent preventive treatment to sulfadoxine pyrimethamine against malaria. Emergent Life Sci Res 4: 53–65.

[26] RBM Malaria Partnership to End Malaria , 2017. Malaria Social and Behavior Change Communication Indicator Reference Guide: Second Edition. Venier, Switzerland: RBM.

[27] Alvarado G Skinner M Plaut D Moss C Kapungu C Reavley N, 2017. A Systematic Review of Positive Youth Development Programs in Low-and Middle-Income Countries. Washington, DC: YouthPower Learning, Making Cents International. 10.1016/j.jadohealth.2019.01.024 31010725

[28] United States Agency for International Development , 2022. *Youth in Development Policy: 2022 Update*. Available at: https://www.usaid.gov/sites/default/files/2022-12/USAID-Youth-in-Development-Policy-2022-Update-508.pdf. Accessed July 6, 2022.

[29] Evans DK Acosta AM, 2020. Lifting bans on pregnant girls in school. Lancet 396: 667–668. 32891208 10.1016/S0140-6736(20)30856-4

