# Supplementary material for: Malaria-Related Psychosocial Factors, Past Antenatal Care–Seeking Behaviors, and Future Antenatal Care–Seeking Intentions by Maternal Age in Malawi and Democratic Republic of the Congo
Source: Am J Trop Med Hyg. 2023 Jun 26;109(2):277–83. doi: 10.4269/ajtmh.23-0069 (PMC10397429; doi:10.4269/ajtmh.23-0069)
Supplement: Supplementary file 1 [file tpmd230069.SD1.pdf]

## SUPPLEMENTAL TABLE 1: IDEATIONAL VARIABLES EXPLORED IN THIS STUDY

| Supplemental Table 1: Prevalence of ideational variables explored in this study | | |
|---|---|---|
| **Survey Question (and Desired Response (%))** | **Malawi (N = 827)** | **DRC (N = 1,312)** |
| **Knowledge of Malaria** | | |
| What signs or symptoms would lead you to think that a person has malaria (fever) | 75 | 87 |
| What causes malaria (Mosquito bites) | 76 | 91 |
| What are the things that people can do to stop them from getting malaria (sleep under an (insecticide-treated) mosquito net, use mosquito repellant, take preventive medication, spray house with insecticide) | 91 | 92 |
| **Knowledge of Early ANC** | | |
| When should a pregnant woman go for pregnancy care for the first time (In the first trimester) | 53 | 36 |
| How many times should a woman go for a prenatal visit during one pregnancy? (4 or more) | 92 | 28 |
| **Attitudes towards Early ANC** | | |
| Even if a woman thinks she may be pregnant, she should wait a few months before she sees a health provider (disagree) | 71 | 40 |
| A woman who has given birth before does not need to see a health provider as soon as she thinks she might be pregnant. (disagree) | 88 | 78 |
| **Perceived Susceptibility to Malaria** | | |
| People in this community only get malaria during rainy season (disagree) | 39 | 50 |
| Nearly every year, someone in this community gets a serious case of malaria (agree) | 85 | 67 |
| **Perceived Severity to Malaria** | | |
| Every case of malaria can potentially lead to death (agree) | 89 | 60 |
| When someone you know gets malaria, you usually expect them to completely recover in a few days (disagree) | 28 | 29 |
| **Perceived Self-efficacy** | | |
| Do you think you could or could not go for antenatal care so soon as I think might be pregnant (could) | 96 | 79 |
| **Recent Interpersonal Communication on ANC** | | |
| When did you last discuss going to a prenatal consultation with your spouse/partner? (ever) | 62 | 56 |
| **Favorable Perceptions of Health Facility Providers in regard to ANC** | | |
| In your community, providers at the health facility make pregnant women pay for | 73 | 75 |

| | | |
|---|---|---|
| SP/Fansidar or Novidar SP (3 pills), the medicine to prevent malaria | | |
| Prenatal health providers in this community generally treat pregnant women with respect (agree) | 73 | 90 |
| **Perceived Supportive Community Norms** | | |
| Generally, how many women in your community go to antenatal care at least four times when they are pregnant? (at least half) | 80 | 74 |

## SUPPLEMENTAL TABLE 2: FULL MODEL OF FACTORS ASSOCIATED WITH INTENTION TO START ANC EARLY IN A FUTURE PREGNANCY

| **Factors associated with intention to start ANC early in future pregnancy** | | | | |
|---|---|---|---|---|
| | **Malawi** | | **DRC** | |
| | **≤20 (N = 234)** | **> 20 (N = 593)** | **≤ 20 (N = 233)** | **> 20 (N = 1,088)** |
| ANC Ideational Score | 1.75 (1.29, 2.38) | 1.52 (1.21, 1.91) | 1.58 (1.18, 2.13) | 1.31 (1.07, 1.60) |
| Attended ANC early in last pregnancy | 2.26 (0.97, 5.25) | 3.60 (1.86, 6.97) | 29.80 (7.81,113.65) | 6.70 (3.89,11.55) |
| **Region** | | | | |
| Northern/ North (Ref) | 1.00 (N/A) | 1.00 (N/A) | 1.00 (N/A) | 1.00 (N/A) |
| Central | 1.07 (0.42, 2.76) | 0.92 (0.44, 1.89) | | |
| Southern | 5.36 (0.98,29.29) | 0.48 (0.20, 1.17) | | |
| West | | | 2.29 (0.71, 7.34) | 2.05 (1.19, 3.53) |
| Kasai | | | 11.30 (3.29,38.78) | 3.15 (1.99, 4.97) |
| Great East | | | 3.44 (0.97,12.14) | 1.49 (0.87, 2.56) |
| **Demographics** | | | | |
| Residence, Rural vs Urban | 1.05 (0.29, 3.83) | 0.30 (0.11, 0.81) | 0.35 (0.12, 0.99) | 0.45 (0.29, 0.71) |
| Education, ≥ Primary vs. < Primary | 1.03 (0.41, 2.59) | 0.94 (0.47, 1.89) | 1.51 (0.45, 5.04) | 1.40 (0.86, 2.30) |
| Wealth quintile, Upper two quintiles vs. lower three | 0.73 (0.28, 1.86) | 0.28 (0.10, 0.83) | 0.97 (0.34, 2.74) | 0.86 (0.53, 1.37) |
| Marital status, Married/Cohabiting vs. single/ divorced | 0.63 (0.17, 2.31) | 0.58 (0.22, 1.53) | 0.67 (0.22, 2.09) | 1.51 (0.92, 2.47) |
| Exposure to malaria message in past six months | 2.19 (0.48,10.02) | 1.27 (0.49, 3.31) | 0.67 (0.22, 2.09) | 1.51 (0.92, 2.47) |
| Parity > 1 | 2.52 (0.66, 9.58) | 1.35 (0.76, 2.38) | 1.03 (0.42, 2.50) | 0.74 (0.42, 1.31) |

*Notes:*p < 0.05, **p < 0.01, ***p < 0.001* [1]
*Reference group is the null (no category), except where specified*
